# Supplementary material for: Bioimage analysis for multiplexed FUCCI acquisitions powered by deep learning
Source: Npj Imaging. 2026 Apr 14;4:27. doi: 10.1038/s44303-026-00159-6 (PMC13079876; doi:10.1038/s44303-026-00159-6)
Supplement: Supplementary file 1 — Supplementary Information [file 44303_2026_159_MOESM1_ESM.docx]

Supplementary Information for:

Bioimage analysis for multiplexed FUCCI acquisitions powered by deep learning

Zimmermann, J., Pezzotti, M., Torchia, E., Enrico, A., Rigolli, S., Di Sante, M., Pasqualini, F.S. *

Synthetic Physiology Lab, Dipartimento di Ingegneria Civile e Architettura, Università di Pavia

* Corresponding author: F.S. Pasqualini, francesco.pasqualini@unipv.it

# HT1080 test dataset (own dataset)

The HT1080 cells were imaged at different levels of confluency with 20x magnification (746 nuclei) and 40x magnification (734 nuclei) (see Fig. S1a and S1b, respectively).
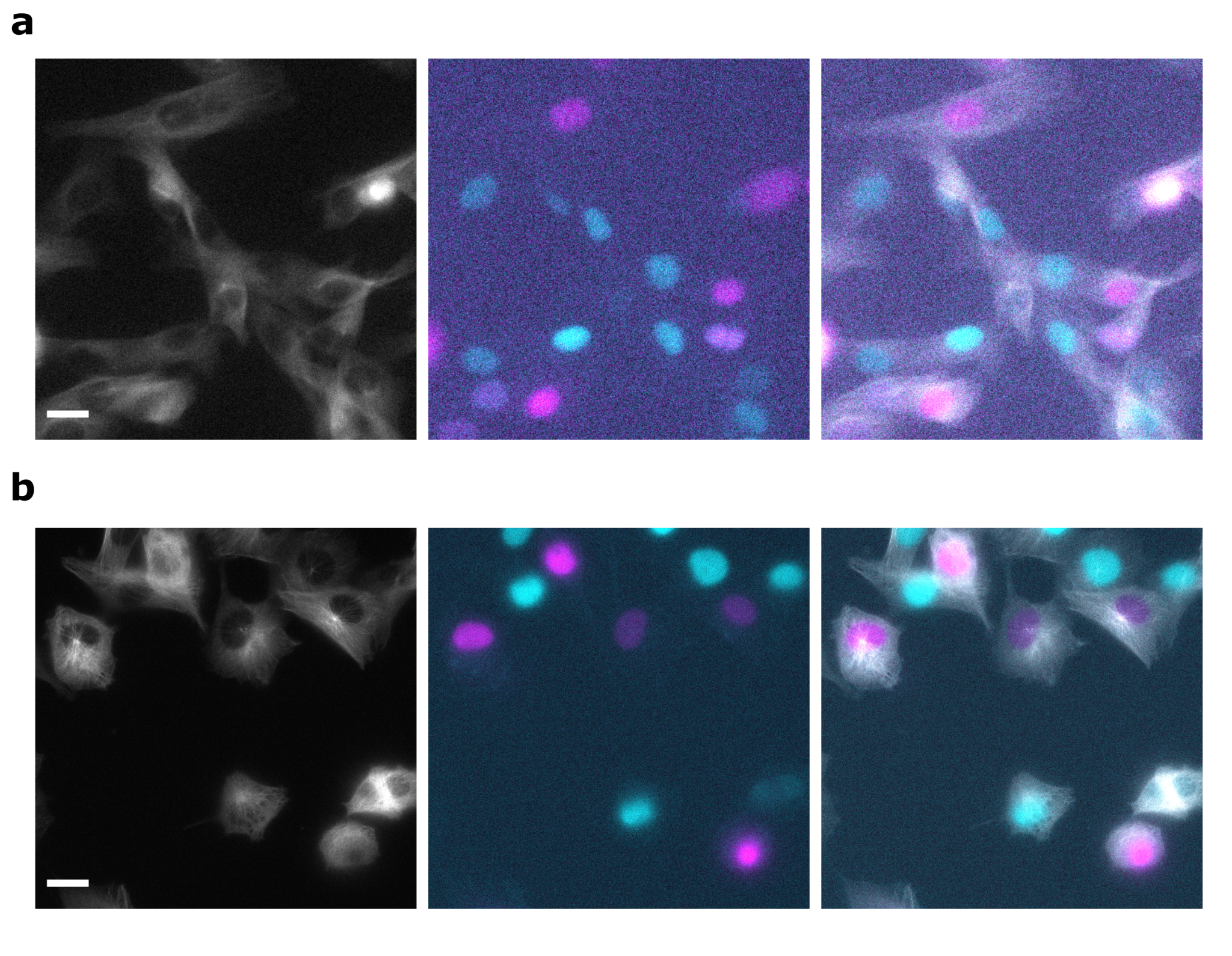
Figure S1: Exemplary HT1080 dataset used to test the segmentation network: Cells were imaged with 20x magnification (**a**) and 40x magnification (**b**). The scale bar is 20 µm.

# Han et al.[^1^](https://www.zotero.org/google-docs/?wmOhaW)

HaCaT cells labelled with FUCCI sensor (Fig. S2a) and SMAD reporter (Fig. S2b), video with in total about 2500 labelled nuclei. This dataset was recorded with a high magnification (100x) and has high signal-to-noise and signal-to-background ratios.


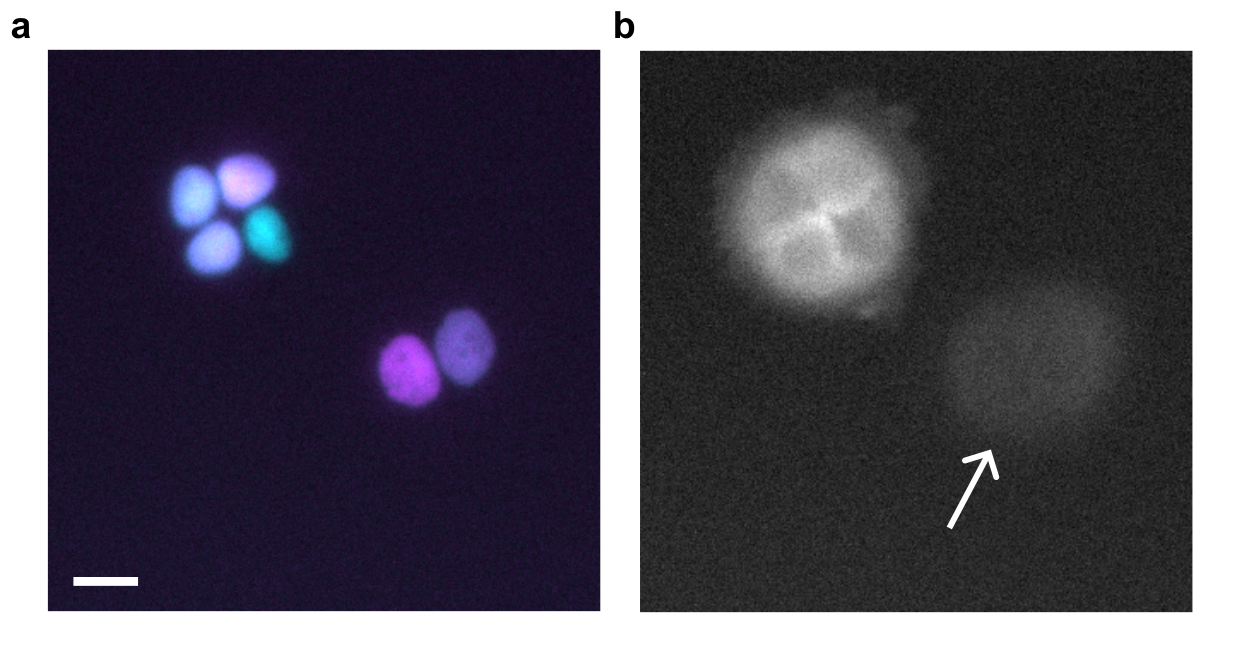
Figure S2: Example from the dataset of Han et al.[^1^](https://www.zotero.org/google-docs/?FKXDUt): **a** FUCCI signal, **b** SMAD signal. The area with low SMAD signal, where the nucleus is not clearly visible is indicated by an arrow. The scale bar is 10 µm.

## ConfluentFUCCI

Example dataset from their GitHub repository (https://github.com/leogolds/ConfluentFUCCI), we used 15 frames out of 60 with about 1000 labelled nuclei.
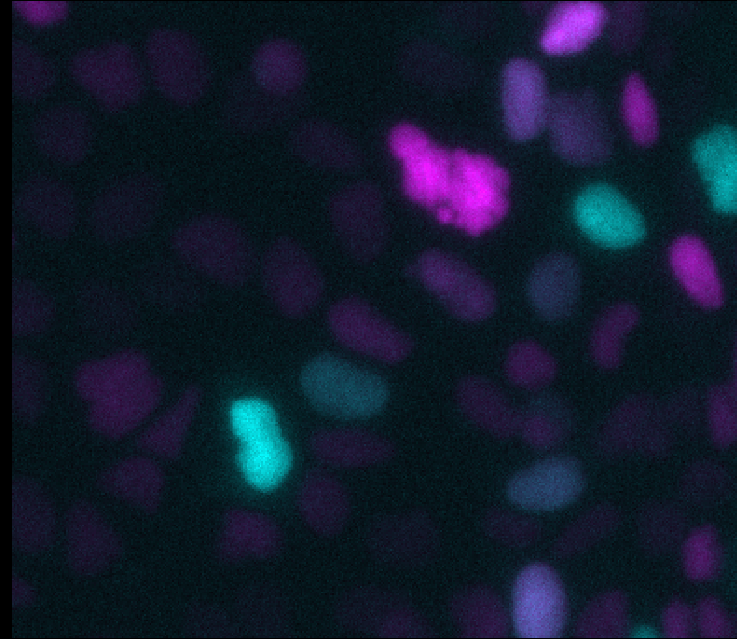

Figure S3: Example frame from the ConfluentFUCCI dataset[^2^](https://www.zotero.org/google-docs/?axrXIk). No scale bar is provided because metadata on pixel size was missing.

## CellMAPtracer

The dataset comprises RPE1-hTert cells labelled with PIP-FUCCI sensor. We labelled one frame containing 275 nuclei. Some of them are not visible (Fig. S4).
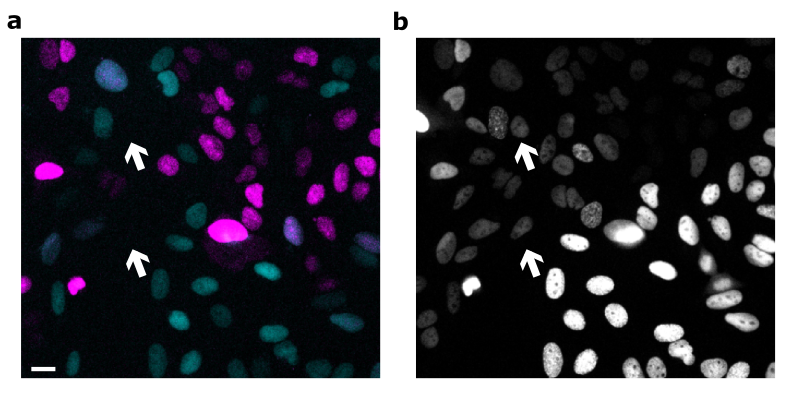


Figure S4: **a** FUCCI signal of the CellMAPtracer data[^3^](https://www.zotero.org/google-docs/?agIMxJ). **B** The nuclei were additionally labelled with PCNA because the PIP-FUCCI signal vanishes between G1 and S phase (see arrows). These nuclei were still included in the dataset and explain the in comparison lower accuracy. The scale bar is 20 µm.

# Cotton et al.

Cotton et al. presented a new PIP-H2A reporter[^4^](https://www.zotero.org/google-docs/?oULvTN). We used one labelled frame with about 1900 labelled nuclei (see an example in Fig. S5).


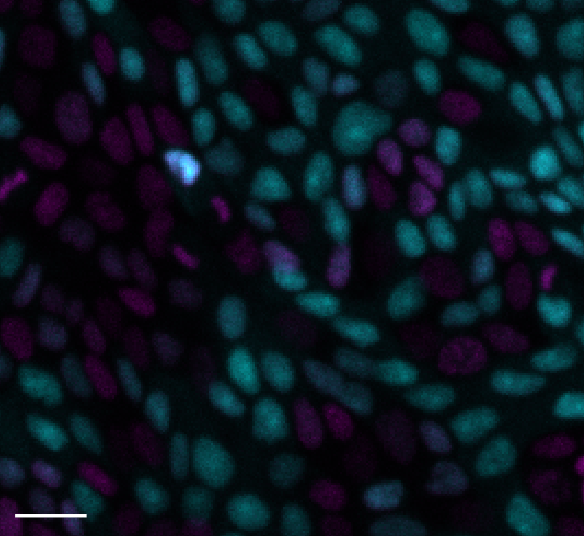


Figure S5: Example FUCCI data from Cotton et al.[^4^](https://www.zotero.org/google-docs/?3B5DGU) The scale bar is 20 µm.

# Classification with Cellpose-SAM


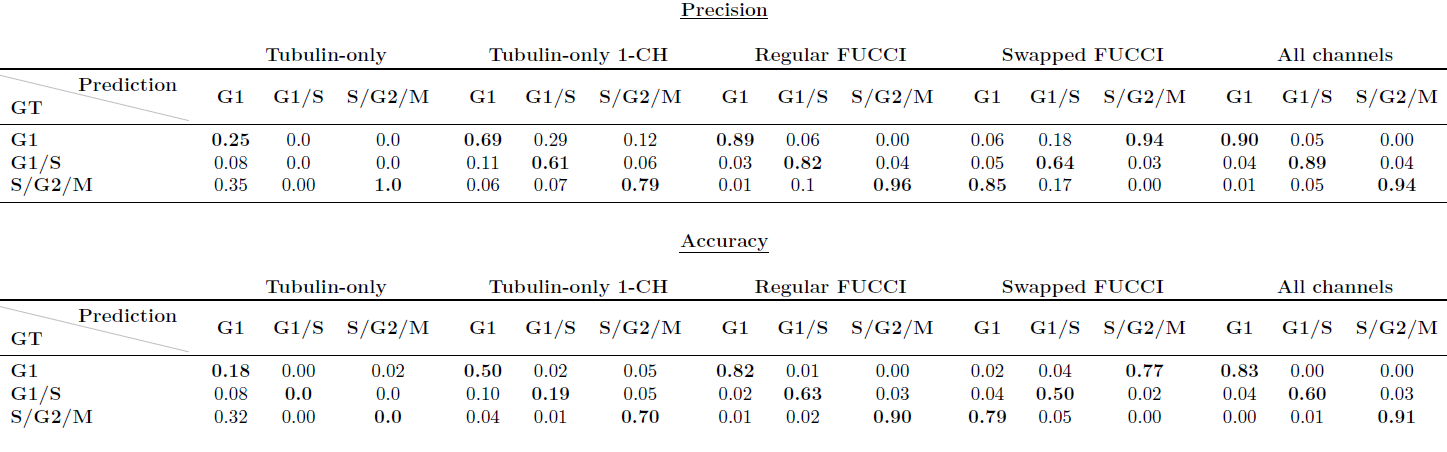


**Table S1** Classification using the Cellpose-SAM network. The network was trained using all channels, only the “Tubulin-only 1CH” configuration was trained on the single tubulin channel. The network is by construction channel-invariant and can be evaluated using an arbitrary number of channels. This Table accompanies Table 2 of the manuscript.

| Color / Status | Magenta ON | Magenta OFF |
| --- | --- | --- |
| Cyan ON | G1/S  G2/M | G1  G1 |
| Cyan OFF | S/G2/M  S | Early G1  Early S |

**Table S2** Color combinations and the corresponding cell cycle phase for the conventional FUCCI sensor[^5^](https://www.zotero.org/google-docs/?lOD2e3) (not underlined) and the PIP-FUCCI sensor[^6^](https://www.zotero.org/google-docs/?eCZi3R) (underlined).

| Available data | **Nuclear segmentation** | **Single-frame classification** | **Pseudotime inference** |
| --- | --- | --- | --- |
| **Tubulin only** | Lower accuracy | Not reliable | Not applicable |
| **FUCCI only** | Misses low intensity nuclei | Reliable | Yields FUCCI intensities |
| **Tubulin and FUCCI** | Detects most nuclei | Reliable | Yields FUCCI intensities, high tracking accuracy |

**Table S3** Summary of the expected results for the respective tasks and the available datasets.

| **Model** | **Pre-trained model** |
| --- | --- |
| I - DAPI-equivalent approach, no pre-processing, pre-trained StarDist segmentation | StarDist |
| II - DAPI-equivalent, post-processing, pre-trained StarDist | StarDist |
| III - DAPI-equivalent, post-processing, pre-trained generalist Cyto3 model | Cellpose Cyto3 |
| IV - DAPI-equivalent model with denoising Cyto3 model | Cellpose Cyto3 with Denoiser |
| V - Pre-trained ConfluentFUCCI, pre-trained channel-invariant InstanSeg with tubulin-only input | Cellpose trained on ConfluentFUCCI data |
| VI - InstanSeg, pre-trained, 1-CH (tubulin-only network) | Channel-invariant InstanSeg |
| VII - InstanSeg, pre-trained, 2-CH (FUCCI channels) | Channel-invariant InstanSeg |
| VIII - InstanSeg, pre-trained, 3-CH (tubulin + FUCCI channels) | Channel-invariant InstanSeg |

Table S4: Comparison of pre-trained benchmark methods


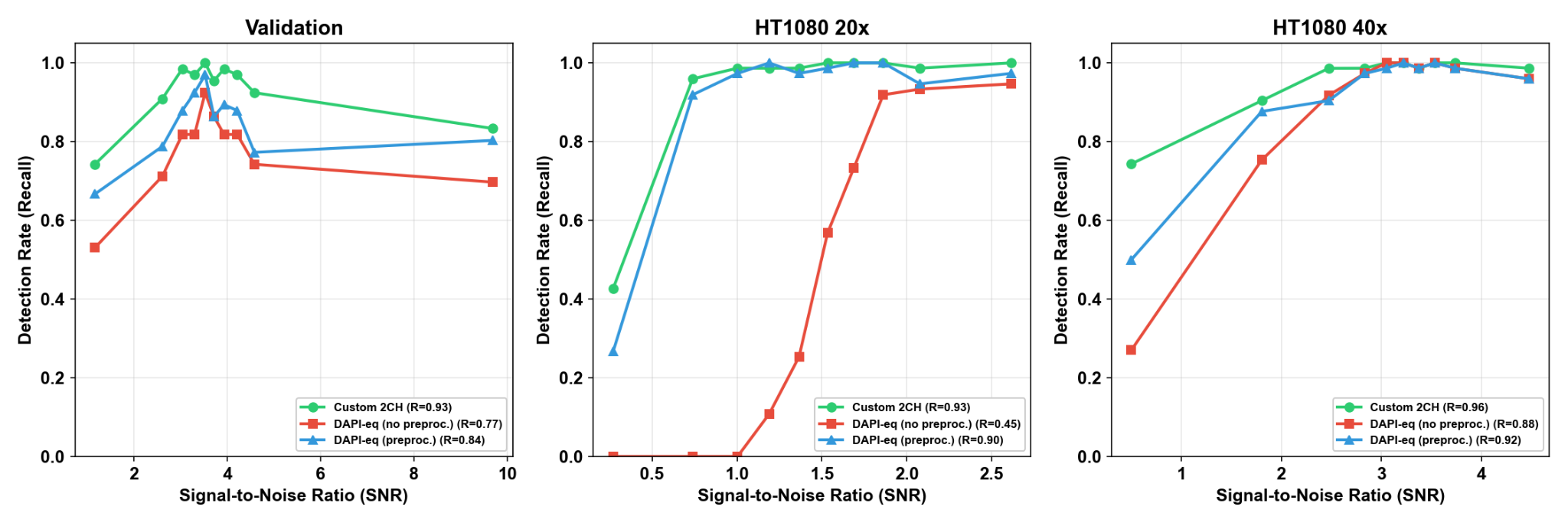


Figure S5: Comparison of the detection rates (recalls) of the custom-trained network against the pretrained DAPI-equivalent (DAPI-eq) methods on the SNR.


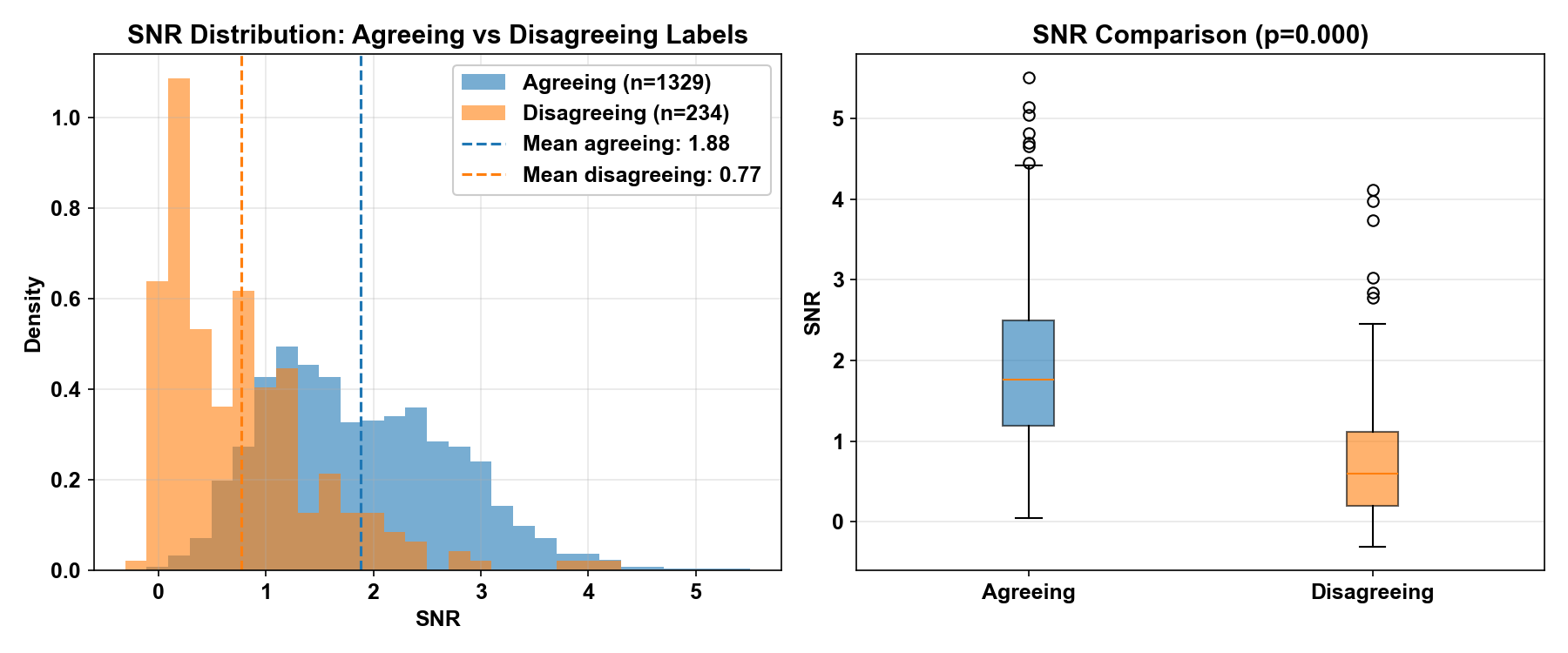


Figure S6: Analysis of SNR for interannotator comparison for both the HT1080 20x and 40x datasets.

# Movies

Movie S1: Comparison of the custom-trained segmentation networks (1-CH, 2-CH, and 3-CH) on a HT1080 example imaged with 20x magnification.

Movie S2: Comparison of the custom-trained classification networks (1-CH, 2-CH, and 3-CH) on a HT1080 example imaged with 20x magnification (same data as shown in Movie S1). The phase is indicated by the color of the bounding box of the individual segmentation masks (cyan -. G1, brown - G1/S, magenta - S/G2/M).

Movie S3: Example of HT1080 cells annotated with cell cycle percentages, movie of data shown in Figure 4a.

Movie S4: Example of HaCaT cells in a scratch assay experiment imaged with 100x magnification, movie of data shown in Figure 4b.

# References

[1.](https://www.zotero.org/google-docs/?8goYx1) [Han, H., Wu, G., Li, Y. & Zi, Z. eDetect: A Fast Error Detection and Correction Tool for Live Cell Imaging Data Analysis. *iScience* **13**, 1–8 (2019).](https://www.zotero.org/google-docs/?8goYx1)

[2.](https://www.zotero.org/google-docs/?8goYx1) [Goldstien, L., Lavi, Y. & Atia, L. ConfluentFUCCI for fully-automated analysis of cell-cycle progression in a highly dense collective of migrating cells. *PLOS ONE* **19**, e0305491 (2024).](https://www.zotero.org/google-docs/?8goYx1)

[3.](https://www.zotero.org/google-docs/?8goYx1) [Ghannoum, S. *et al.* CellMAPtracer: A User-Friendly Tracking Tool for Long-Term Migratory and Proliferating Cells Associated with FUCCI Systems. *Cells* **10**, 469 (2021).](https://www.zotero.org/google-docs/?8goYx1)

[4.](https://www.zotero.org/google-docs/?8goYx1) [Cotton, M. J. *et al.* An in vitro platform for quantifying cell cycle phase lengths in primary human intestinal epithelial cells. *Sci. Rep.* **14**, 15195 (2024).](https://www.zotero.org/google-docs/?8goYx1)

[5.](https://www.zotero.org/google-docs/?8goYx1) [Sakaue-Sawano, A. *et al.* Visualizing Spatiotemporal Dynamics of Multicellular Cell-Cycle Progression. *Cell* **132**, 487–498 (2008).](https://www.zotero.org/google-docs/?8goYx1)

[6.](https://www.zotero.org/google-docs/?8goYx1) [Grant, G. D., Kedziora, K. M., Limas, J. C., Cook, J. G. & Purvis, J. E. Accurate delineation of cell cycle phase transitions in living cells with PIP-FUCCI. *Cell Cycle* **17**, 2496–2516 (2018).](https://www.zotero.org/google-docs/?8goYx1)
